# Resistance of *Xanthomonas oryzae* pv. *oryzae* to Lytic Phage X2 by Spontaneous Mutation of Lipopolysaccharide Synthesis-Related Glycosyltransferase

**DOI:** 10.3390/v14051088

**Published:** 2022-05-18

**Authors:** Muchen Zhang, Jiahui Qian, Xinyan Xu, Temoor Ahmed, Yong Yang, Chenqi Yan, Mohsen Mohamed Elsharkawy, Mohamed M. Hassan, Jamal A. Alorabi, Jianping Chen, Bin Li

**Affiliations:** 1 State Key Laboratory of Rice Biology and Ministry of Agriculture Key Laboratory of Molecular Biology of Crop Pathogens and Insects, Institute of Biotechnology, Zhejiang University, Hangzhou 310058, China; 11816060@zju.edu.cn (M.Z.); 22016076@zju.edu.cn (J.Q.); 12016074@zju.edu.cn (X.X.); temoorahmed@zju.edu.cn (T.A.); 2 State Key Laboratory for Managing Biotic and Chemical Threats to the Quality and Safety of Agro-Products, Institute of Virology and Biotechnology, Zhejiang Academy of Agricultural Sciences, Hangzhou 310021, China; yangyong@zaas.ac.cn (Y.Y.); yanchengqi@zaas.ac.cn (C.Y.); 3 Institute of Biotechnology, Ningbo Academy of Agricultural Sciences, Ningbo 315040, China; 4 Agricultural Botany Department, Faculty of Agriculture, Kafrelsheikh University, Kafr El-Sheikh 33516, Egypt; mohsen.abdelrahman@agr.kfs.edu.eg; 5 Department of Biology, College of Science, Taif University, Taif P.O. Box 21974, Saudi Arabia; m.khyate@tu.edu.sa (M.M.H.); dr.jamal.a@gmail.com (J.A.A.); 6 State Key Laboratory for Managing Biotic and Chemical Threats to the Quality and Safety of Agro-Products, Institute of Plant Virology, Ningbo University, Ningbo 315211, China

**Keywords:** *Xanthomonas oryzae* pv. *oryzae*, phage resistance, virulence, lipopolysaccharide, glycosyltransferase

## Abstract

Phage therapy is a promising biocontrol management on plant diseases caused by bacterial pathogens due to its specificity, efficiency and environmental friendliness. The emergence of natural phage-resistant bacteria hinders the application of phage therapy. *Xanthomonas oryzae* pv. *oryzae* (Xoo) is the causal agent of the devastating bacterial leaf blight disease of rice. Here, we obtained a spontaneous mutant C2R of an Xoo strain C2 showing strong resistance to the lytic phage X2. Analysis of the C2R genome found that the CDS2289 gene encoding glycosyltransferase acquired a frameshift mutation at the 180th nucleotide site, which also leads to a premature stop mutation at the 142nd amino acid. This mutation confers the inhibition of phage adsorption through the changes in lipopolysaccharide production and structure and bacterial surface morphology. Interestingly, glycosyltransferase-deficient C2R and an insertional mutant k2289 also showed reduced virulence, suggesting the trade-off costs of phage resistance. In summary, this study highlights the role of glycosyltransferase in interactions among pathogenic bacteria, phages and plant hosts, which provide insights into balanced coevolution from environmental perspectives.

## 1. Introduction

Bacterial leaf blight (BLB), caused by *Xanthomonas oryzae* pv. *oryzae* (Xoo), is one of the most destructive diseases of rice crops and occurs in most rice-growing areas worldwide [1]. Due to the increased environmental concern, efficient and environmentally friendly biocontrol managements including the application of antagonistic bacteria against Xoo and phage therapy are attracting more attention to control this rice bacterial disease [2,3,4]. In nature, Xoo becomes resistant to phage via spontaneous mutation. No information is available about the phage-resistance of Xoo by spontaneous mutants, which greatly limits phage therapy against bacterial infection in rice [5,6].

The successful infection of phage to bacterial hosts depends on the binding of phage to host cell surface receptors, which include outer membrane proteins, exopolysaccharide, capsular polysaccharide, cell wall teichoic acids, lipopolysaccharide (LPS) and other bacterial cell appendages, such as flagella and pili [1,7,8,9,10,11]. Among these receptors, LPS, the major component of the outer membrane, is associated with phage adsorption [12], virulence to eukaryotic hosts [8,13] and environmental adaptation [7,14]. Furthermore, the role of LPS in phage infection was revealed by knockout mutation of LPS encoding-related genes, which causes resistance to phage infection by the abortion of phage adsorption [15,16]. These results highlight the importance of LPS in the interaction between phage and host bacteria.

In a natural environment, spontaneous mutation [17] is the main driving force of coevolution between phage and host bacteria [6]. Spontaneous mutations cause bacterial resistance to phage at various stages of phage infection [5], in particular, the abortion of phage adsorption [18], which is the first step of phage infection for host bacteria. In order to effectively utilize the phages in plant disease control, it is necessary to explore the mechanism of phage-resistance mutation under natural conditions.

Phage X2 was isolated from diseased rice leaves in China and belongs to the family *Myoviridae*, possessing icosahedral heads, necks and base plates with tail fibers. Phage X2 exhibited strong lytic activity against the Xoo virulent strain C2 [19]. Recently, one spontaneous mutant C2R of Xoo C2 showing strong resistance to phage X2 was screened out. The genetic background of this mutation is not clear.

The purpose of this study is to understand the mechanism of bacterial phage resistance by identifying the mutated gene of the spontaneous mutant C2R that impairs the infection of phage X2 to Xoo based on phenotypic and genomic analysis and molecular studies. The role of the mutated gene in phage–Xoo interactions was investigated by comparing the phage adsorption, LPS synthetization and the pathogenicity to rice seedlings between the wild type and spontaneous mutant C2R, which was further verified by studying an insertional mutant and the corresponding complement of Xoo strain C2.

## 2. Materials and Methods

### 2.1. Bacterial Strains, Phages, and Media

Nutrient broth (NB) or nutrient agar (NA) were used to culture Xoo strain C2 and its lytic phage X2 at 30 °C through shaking or plate culturing, while *Escherichia coli* strains were grown in Luria–Bertani (LB) broth or LB agar at 37 °C supplemented with appropriate antibiotics [20] (Table 1). The wild-type Xoo strain C2 was used as a host to proliferate phage X2, which was isolated from diseased rice leaves as previously described [19].

### 2.2. Phage Propagation Assays

Phage X2 propagation was conducted according to the phage lysate method [24,25]. In brief, 50 μL of phage (10^10^ PFU/mL) was added to a 500 μL overnight bacterial culture (10^8^ CFU/mL) and was shaken at 30 °C for 6 to 8 h until the culture was cleared. After the centrifugation at 10,000× *g*, 4 °C for 20 min and filtration through a 0.22 μm PES membrane filter (Merck Millipore Ltd., Carrigtwohill, Ireland), the phage lysate was collected and stored at 4 °C until use. Lysis activity of phage X2 on Xoo strains was examined using spot assay by mixing 500 μL of exponential-phase Xoo cultures and 5 mL of molten NA with 0.8% (*w*/*v*) of agar and pouring the mixture into NA plates to produce double agar plates. Then, 2 μL of 10-fold serial dilution of phage was spotted onto the surface of the plates. After overnight incubation at 30 °C, the phage plaque was examined and photographed.

### 2.3. Isolation of Phage-Resistant Mutants

Spontaneous phage-resistant mutants were screened out using the secondary culture method [26]. Briefly, after the phage X2 infection on an overnight culture of wild-type Xoo strain C2 at 30 °C for 6 to 8 h to complete cell lysis, secondary growth was conducted though further incubation for up to 48 h at 30 °C until the bacterial culture became turbid again. Then, the phage-resistant mutants were cultured and purified by consecutive streaking on NA plates at 30 °C, and the identity of the Xoo mutants was confirmed by amplification and analysis of the 16S rRNA gene. Furthermore, the obtained phage-resistant mutants were confirmed by examining their capacities in plaque formation, phage-resistance stability and adsorption.

### 2.4. Genome Analysis of Phage-Resistant Mutants

After obtaining phage-resistant mutants, one mutant designated as strain C2R was selected for genome sequencing according to the previously described method [27]. Briefly, the C2R genome was sequenced using Illumina NovaSeq PE150. Genomic alignment was conducted between the sample genome C2R and reference genome C2; SNP (Single Nucleotide Polymorphism), indel (insertion and deletion) and SV (Structural Variation) were performed using the MUMmer [28] and LASTZ [29] tools. Furthermore, in order to examine the conservation of the mutated genes, protein sequences of these genes from various bacteria were downloaded through UniProt. After sequence alignment using ClustalW software, the phylogenetic tree of mutated gene was constructed by the maximum likelihood method using Molecular Evolutionary Genetics Analysis (MEGA) (version 6.0, Mega Limited, Auckland, New Zealand) [30].

### 2.5. Construction and Complementation of Mutants

In order to further confirm the function of the mutated gene against phage X2 in Xoo strain C2, the insertional and complement mutants of the selected genes were generated as described in previous studies [22,23] through homologous recombination using suicide plasmid pJP5603 and pRADK, respectively. In brief, the recombinant plasmid pJP5603 or pRADK were constructed and electro-transformed at 2200 V, 25 μF, 400 Ω. After the screening of Km and Km+ Cm resistance, the insertional and the complemented mutants were picked up, respectively, which were further identified through PCR amplification using site-specific primers (Table 2).

### 2.6. Quantitative Real-Time RT-PCR

The role of mutated gene related to the resistance of Xoo C2 to phage X2 was further determined by investigating the expression of this gene in the wild-type C2, spontaneous phage-resistant mutant C2R, insertional mutant k2289 and complement c2289, which was carried out using quantitative real-time RT-PCR (qRT-PCR) [31]. Briefly, total RNAs of bacterial strains were extracted using RNAiso (Takara Biotechnology Co. Ltd., Dalian, China). Afterwards, cDNAs were synthesized by using the HiScript II Q RT supermix for qPCR (+gDNA wiper) (Vazyme Biotechnology Co. Ltd., Nanjing, China). qRT-PCR was conducted on an ABI PRISM 7500 Sequence Dectection System using ChamQ SYBR qPCR Master Mix Q311-02 (Vazyme Biotechnology Co. Ltd., Nanjing, China) following the manufacturer’s protocol. Data analysis was conducted through the comparative threshold cycle method and the reference gene *gyrB* was used for normalization. The relative expression level is calculated and shown as the ratio to that of the wild type. The experiment was repeated three times and carried out in triplicates.

### 2.7. Phage Adsorption Assays

The adsorption efficiency of phage X2 to Xoo strains was determined by mixing 50 μL of phage X2 (approximately 10^10^ PFU/mL) with 500 μL of exponential-phase cultures (10^8^ CFU/mL) of Xoo strains, incubating at 30 °C for 30 min for fully adsorption, and then centrifuging at 13,000× *g* for 5 min. In order to determine the unabsorbed phage, the phage titers remaining in the supernatant were evaluated based on the assay of double layer plates with Xoo strain C2. The adsorption efficiency was calculated by dividing the adsorbed phage number by initial phage number [16]. Each assay was conducted three times.

### 2.8. Electron Microscopic Observation

Electron microscopy on Xoo was conducted according to the previous method [25]. In short, after infection with phage X2 at multiplicity of infection (MOI) = 1:100, bacterial cultures were collected immediately (0 h) or after 6 h incubation at 30 °C. TEM and SEM samples were prepared following the corresponding protocol.

Furthermore, the response of bacteria to phage infection was investigated with fluorescent Live/Dead BacLight Bacterial viability stain (Molecular Probes, Invitrogen) containing SYTO 9 (green fluorescent, for live bacteria) and propidium iodide (red fluorescent, for dead bacteria) according to the manufacturer’s instructions [25,32]. Inverted confocal microscope LSM780 (Carl Zeiss Microscopy GmbH, Jena, Germany) was used to detect fluorescence. In addition, phage DNA labeling and adsorption assay was conducted as described previously [15] with some modifications. In brief, 100 μL of phage X2 was incubated with 0.5 μM of nucleotide dye STYO BC Green (Invitrogen) for 10 mins at room temperature following the manufactural instructions. After removing the free SYTO BC Green using Millipore Amicon^®^ Ultra-0.5 3K Centrifugal Filter Devices (Merck Millipore Ltd., Carrigtwohill, Ireland) (UFC500396, 14,000× *g* for 10–30 min, then perform reverse spin immediately at 1000× *g* for 2 min), the labeled phages were incubated with bacteria at room temperature for 20 min and monitored based on confocal microscopic observation of the labeled phage DNA.

### 2.9. Assays of LPS

LPS was extracted from 5 mL of different Xoo cultures at the same concentration of OD_600_ = 0.8 using LPS extraction kit (BestBio Biotechnology Ltd., Shanghai, China). Quantification of LPS was conducted by anthrone-sulfuric method [33] with modifications. Briefly, the standard curve of saccharide was generated using anhydrous glucose, the extracted LPS (200 μL) was mixed with anthrone reagent (600 μL) (2 g/L anthrone prepared with concentrated sulfuric acid), and then the reaction mixture was heated in a water bath at 95 °C for 10 min. After cooling down to room temperature, LPS production was determined by measuring the OD value at 625 nm. Furthermore, an LPS profile assay was performed on 12% SDS-polyacrylamide gel electrophoresis (PAGE) and visualized by silver staining as previously described [7].

### 2.10. Assay of Hypersensitive Response (HR) and Rice Seedlings Pathogenicity

To determine HR to Xoo by nonhost tobacco (*Nicotiana tabacum*), overnight Xoo culture adjusted to OD_600_ = 0.8 was washed and infiltrated into tobacco leaves using a 1 mL syringe without a needle as previously described [34]. HR lesions were observed at 24 h after inoculation. The pathogenicity of bacteria to rice seedlings was determined on 3-week-old leaves of rice seedlings (cultivar quanliangyou 1606) in a greenhouse (16 h day/8 h night; 28 °C) by observing disease symptoms and measuring lesion lengths at 14 d after bacterial inoculation using the scissor clip method [35].

### 2.11. Statistical Analysis

All experiments were repeated at least three times. Statistical analysis was performed with SPSS (IBM Statistics for Windows, Version 19.0. Armonk, NY, USA) software and analyzed by one-way analysis of variance (ANOVA). Comparison of means was carried out using Duncan’s multiple range test at the level of *p* ≤ 0.05.

## 3. Results

### 3.1. Spontaneous Mutant C2R Showed Clear Resistance to Phage X2

Spontaneous mutants resistant to phage X2 were screened out using the secondary culture method. One representative mutant showing strong resistance to phage X2 infection was designated as Xoo strain C2R and selected for further study.

**Figure 1:**
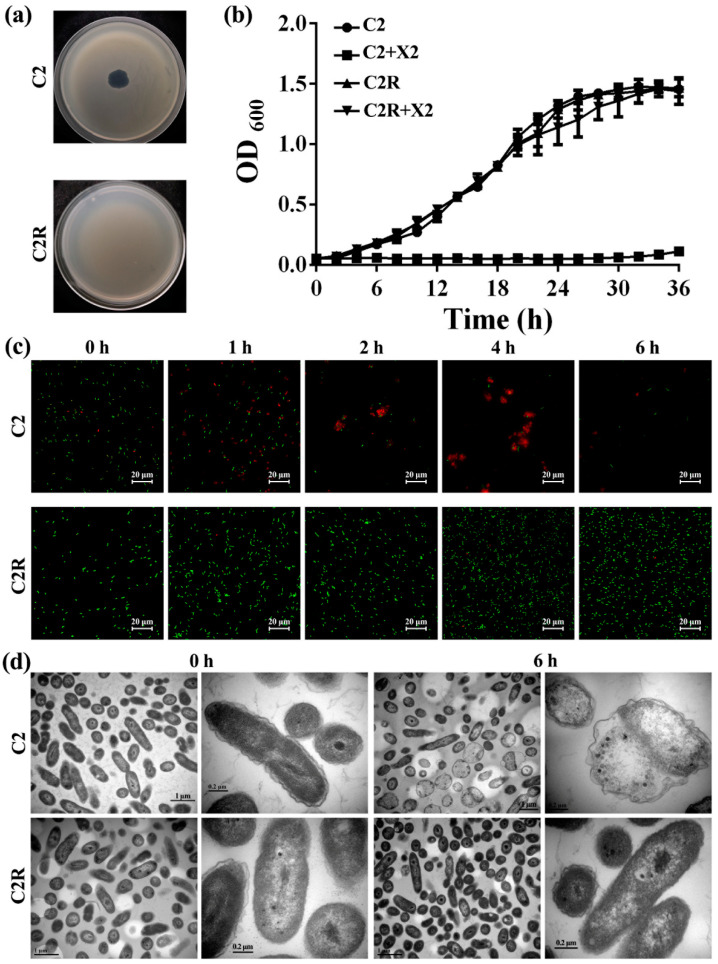
Resistance of Xoo spontaneous mutant C2R to phage infection. (**a**) Spot assays on Xoo wild-type strain C2 and mutant C2R by spotting 2 μL of phage solutions onto freshly bacterial lawns and then incubating overnight at 30 °C. (**b**) Bacterial growth curve of Xoo wild-type strain C2 and mutant C2R in presence or absence of phage X2. (**c**) Live/dead cell staining of Xoo wild-type strain C2 and mutant C2R after 0, 1, 2, 4 and 6 h incubation with phage X2. Red and green fluorescent represents dead and live bacteria, respectively. (**d**) TEM images of Xoo wild-type strain C2 and mutant C2R after 0 and 6 h incubation with phage X2.

A phage plaque assay showed that a clear plaque appeared after incubating the phage X2 with the wild-type strain C2, while no plaque was formed with the spontaneous mutant C2R (Figure 1a). Measurement of the growth curve showed that the OD_600_ value of Xoo strain C2 was 0.05, 0.17, 0.40, 0.83, 1.32, 1.45 and 1.45 after 0 h, 6 h, 12 h, 18 h, 24 h, 30 h and 36 h of incubation, respectively, while the addition of phage X2 caused a 4%, 65%, 87%, 94%, 96%, 96% and 92% reduction in the OD_600_ value of Xoo strain C2 after 0 h, 6 h, 12 h, 18 h, 24 h, 30 h and 36 h of incubation, respectively. However, compared to the wild-type Xoo strain C2, the OD_600_ value was unaffected by the spontaneous mutant C2R regardless of the presence or absence of phage X2 after incubation. The growth curve was almost the same as the wild type, whose OD_600_ value kept rising from 0.05 at 0 h to 1.45 at 36 h (Figure 1b).

In agreement with the result of bacterial growth measurement, living/dead bacterial staining indicated that the live cells in green fluorescence of strain C2 were drastically reduced in number with the increase of the incubation time after mixing bacteria with phage X2, leading to the continuous decline of the survival rate from 96.99% (0 h) to merely 0.78% (6 h). In contrast, the cell number of C2R was unaffected and even increased with extended incubation time after mixing bacteria with phage X2 (Figure 1c). In addition, TEM observation indicated that the intact cells of the wild-type strain C2 at 0 h after incubation with phage X2 were damaged or destroyed at 6 h after inoculation, while the cells of mutant C2R were kept intact (Figure 1d).

### 3.2. Genomic Analysis Identified Mutated Gene Encoding Glycosyltransferase

To determine the genomic changes associated with phage resistance, a whole genome resequencing was carried out on spontaneous phage-resistant mutant C2R [36]. Genetic variant analysis on SNPs, indels and SVs, revealed 5 indels and 1 SV (Table 3) passed the filter among 5096 ORFs in the 4,927,243 bp genome, while no SNPs were found. Among the 5 indels, only 1 was located in the coding region of glycosyltransferase, which was selected for further analysis, while others were located in the upstream or downstream of genes encoding mobile element protein, ABC transporter, cold shock protein and avirulence protein, indicating genes encoding these proteins may be involved in bacterial resistance to phage infection.

**Figure 2:**
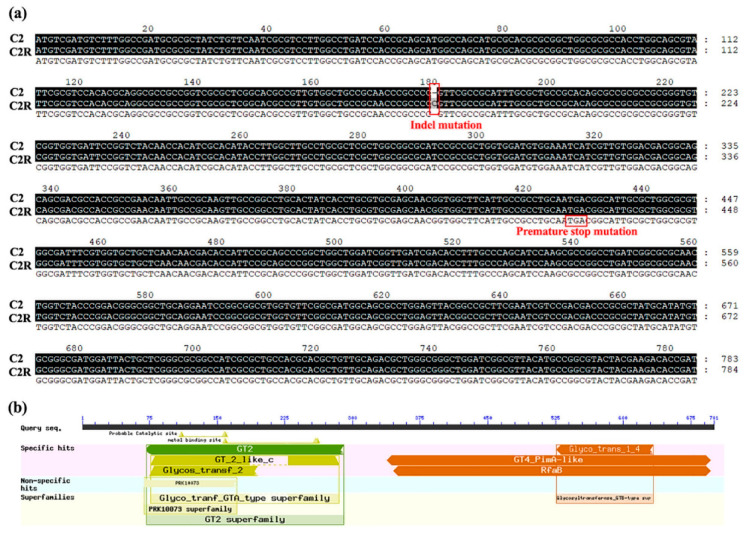
Information about the mutation site CDS2289 of phage-resistant mutant C2R of Xoo. (**a**) Frameshift mutation site and premature stop mutation in mutant C2R. (**b**) Annotation of CDS2289 protein.

Among these mutated genes, we noticed that there was a frameshift mutation at the 180th nucleotide site (CCG→CCCG) in CDS2289 (Figure 2a), which encodes the glycosyltransferase (Figure 2b), resulting in a premature stop codon in translation. The premature stop mutation within CDS2289 accounted for a truncation of 558 (from 142 to 700) amino acids in glycosyltransferase. Furthermore, this mutation site was further verified through PCR amplification (data not shown).

**Figure 3:**
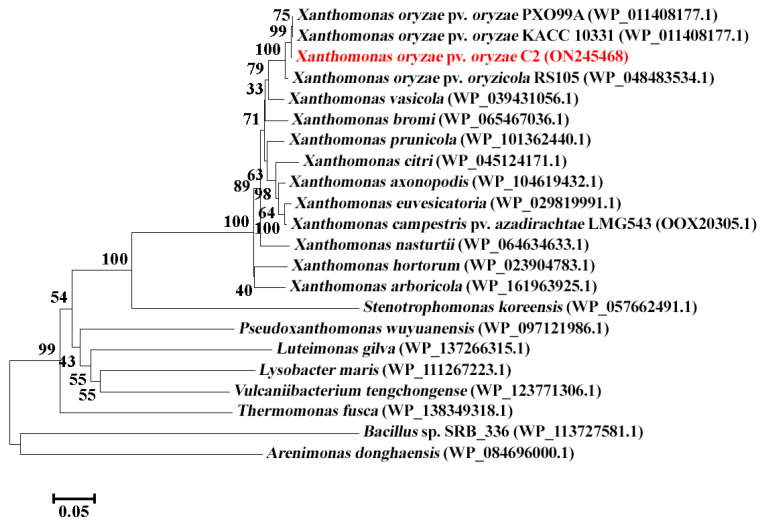
CDS2289 is widely distributed in bacteria. Phylogenetic analysis of CDS2289 glycosyltransferase proteins from different bacteria. The phylogenetic tree was constructed by MEGA 6.0 using the neighbor-joining method. The other 21 glycosyltransferase sequences were collected from UniProtKB. CDS2289 in Xoo wild type C2 is shown in red.

Through multiple alignment, a phylogenetic tree (Figure 3) of CDS 2289 glycosyltransferase proteins of various bacteria was constructed, and the result revealed the close relationship of Xoo C2 CDS 2289 glycosyltransferase to related strains, suggesting this protein is conserved in *Xanthomonas* and widespread in various Gram-negative and -positive bacteria. Interestingly, glycosyltransferase has been found to be required for the biosynthesis of the cell wall including some potential phage receptors such as peptidoglycan and LPS [37]. Therefore, it can be inferred that the mutation in CDS2289 is related with phage resistance.

### 3.3. Verification by Constructing Insertional Mutant and Complement

**Figure 4:**
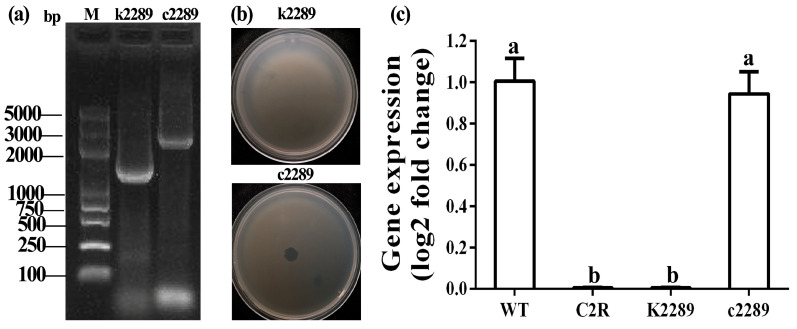
Verification of glycosyltransferase gene in phage resistance by constructing insertional mutant k2289 and complement c2289. (**a**) PCR verification of k2289 and c2289. (**b**) Spot assays of phage X2 on k2289 and c2289. (**c**) Expression of glycosyltransferase gene by qRT-PCR. Data were presented as means ± standard errors (*n* = 3). Columns with different letters are significantly different according to LSD test (*p* < 0.05).

In order to elucidate the roles of CDS2289 gene in bacterial resistance to phage infection, the insertional mutant k2289 was constructed. Furthermore, the constructed mutant k2289 was justified based on PCR amplification of target gene by observing a single band in a 1.5% agarose gel with the expected size of about 1549 bp. To avoid the mutant caused by a polar effect or other spontaneous mutations in bacterial genome, the k2289 mutant was complemented by the corresponding wild-type gene. The constructed corresponding complement c2289 of k2289 was confirmed by observing a single band in a 1.5% agarose gel with the expected size of 3113 bp (Figure 4a).

There was a difference in phage-resistant ability between k2289 and c2289 (Figure 4b). Indeed, phage X2 susceptibility indicated that there was no plaque in k2289, but a small and clear plaque was observed in c2289. This result indicates that insertional mutant lost its sensitivity to phage X2, while the complement strain c2289 partially restored the sensitivity to phage X2 even that the plaque was smaller than that of the wild-type strain. Furthermore, the role of mutated glycosyltransferase gene was identified by examining its expression in C2, C2R, k2289 and c2289. The qRT-PCR results showed that expression of the CDS2289 gene was almost identical between the wild-type strain and c2289, but almost disappeared in C2R and k2289 (Figure 4c).

### 3.4. Mutation of Glycosyltransferase Gene Reduces Phage Adsorption

**Figure 5:**
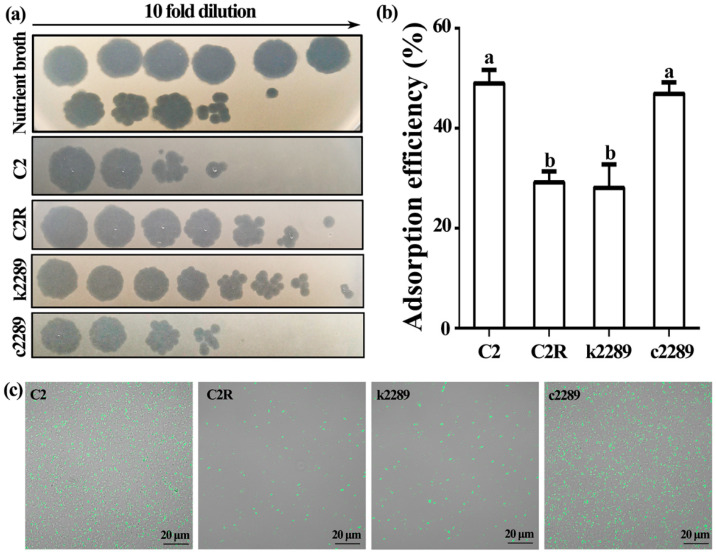
Phage adsorption to wild-type Xoo strain C2, spontaneous phage-resistant mutant C2R, insertional mutant k2289 and complement c2289 after 30 min of incubation. (**a**) Infection of tenfold dilution unabsorbed phage X2 to bacteria. (**b**) Adsorption efficiency of X2 binding to bacteria. Data were presented as means ± standard errors (*n* = 3). Columns with different letters are significantly different according to LSD test (*p* < 0.05). (**c**) Visualization of SYTO-labeled X2 phage adsorption to bacterial surfaces under a laser scanning confocal microscope.

To determine the role of glycosyltransferase gene in phage resistance, the phage adsorption assay was carried out on the wild-type C2, spontaneous phage-resistant mutant C2R, insertional mutant k2289 and complement c2289 (Figure 5a). About 50% of the phages were rapidly adsorbed onto host cells of the wild-type C2 and complement c2289 after 30 min of incubation. In contrast, only about 29% and 28% of the phages were adsorbed onto the host cells of k2289 and C2R, respectively (Figure 5b). The results indicated that phage adsorption to receptors on the bacterial surface of the two mutants was inhibited, suggesting that the mutation of the glycosyltransferase gene altered the phage receptors. The reduction of adsorption efficiency was further confirmed by visualization of SYTO 9 green fluorescent-labeled phage adsorption to bacterial surfaces. Laser scanning confocal microscopy showed that fluorescent-labeled X2 phages heavily attached to the cells of the wild type and the complement c2289 after inoculation, whereas only a small portion of phages attached to C2R and k2289 (Figure 5c).

### 3.5. Mutation of Glycosyltransferase Gene Alters the Bacterial Surface Morphology and Motility

**Figure 6:**
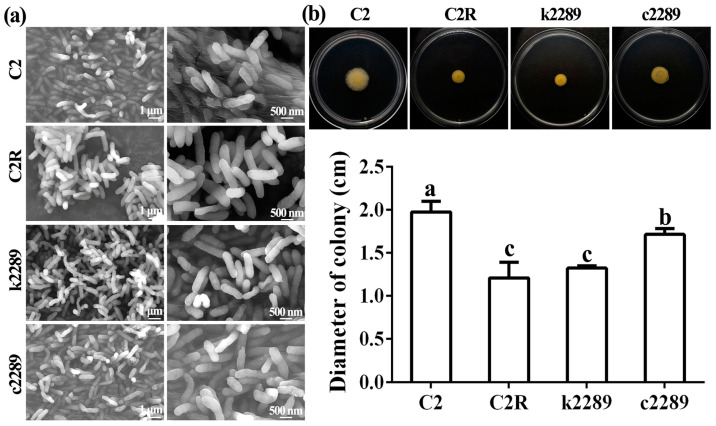
Mutation of glycosyltransferase gene alters the bacterial morphology and motility. (**a**) SEM images about cell surface morphology of the C2, C2R, k2289 and c2289. (**b**) Motility of C2, C2R, k2289 and c2289. Colony diameter (cm) was presented as means ± standard errors (*n* = 3). Columns with different letters are significantly different according to LSD test (*p* < 0.05).

Based on the phage adsorption assay, we speculated that cell surface structure changes of bacterial mutants may result in the inhibition of phage adsorption. To confirm this hypothesis, SEM on bacterial morphology showed that the boundaries of the phage-resistant mutant C2R and insertional mutant k2289 were clearer than those of the wild-type C2 and complement c2289 (Figure 6a). Moreover, bacterial motility of the two mutants C2R and k2289 was weaker than that of the wild-type C2 and complement c2289 (Figure 6b). Xoo strain C2 swam in the semi-solid NA and formed halo with a diameter of 1.98 cm after 48 h of incubation. Bacterial colony diameter was almost identical in complement c2289 but was significantly reduced in C2R and k2289 to 1.21 and 1.32 cm, respectively, after 48 h of incubation. In addition, the bacterial colony were dryer in the two mutants than that of the wild-type C2 and complement.

### 3.6. Mutation of Glycosyltransferase Gene Inhibits Phage Infection by Changing LPS Structure

**Figure 7:**
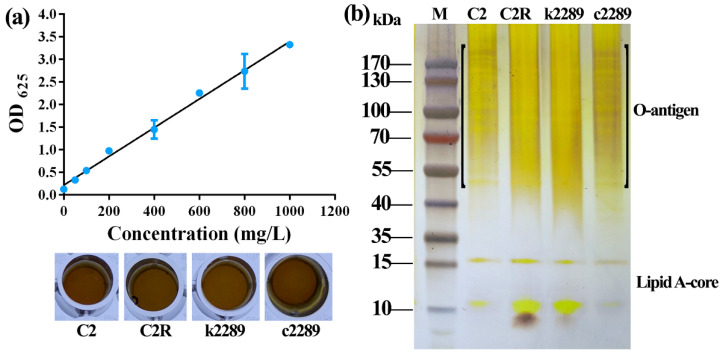
Mutation of glycosyltransferase gene inhibits phage infection by changing LPS structure of Xoo. (**a**) LPS concentration extracting from the bacterial culture at OD_600_ = 0.8 was determined based on the standard curve of saccharide established using glucose and the anthrone-sulfuric acid method. (**b**) LPS profiles of C2, C2R, k2289 and c2289 displayed by SDS-PAGE and silver staining.

To determine if the frameshift and premature stop mutation of CDS2289 influence the synthesis of LPS, we compared the LPS profiles of the wild-type C2, spontaneous phage-resistant mutant C2R, insertional mutant k2289 and complement c2289. The concentration of the LPS extracting from C2, C2R, k2289 and c2289 was 298, 184, 189 and 226 mg/L, respectively. The LPS from C2R and k2289 was reduced 38% and 37%, respectively, while the LPS from c2289 was reduced 24% comparing with the wild-type C2 (Figure 7a). The LPS profiles from C2 and c2289 were clear and similar whereas those from C2R and k2289 appeared blurred particularly in the region of O-antigen (Figure 7b).

### 3.7. Mutation of Glycosyltransferase Gene Reduces Xoo Virulence

**Figure 8:**
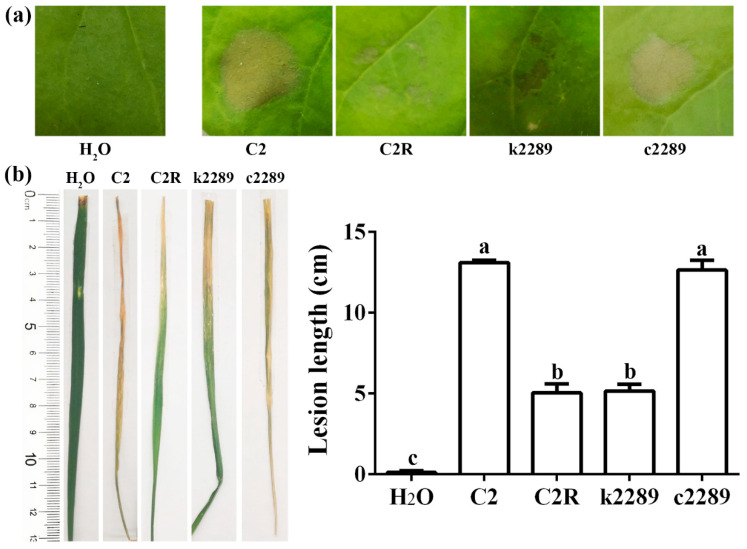
Role of glycosyltransferase gene in Xoo virulence. (**a**) HR lesions in tobacco leaves determined at 24 h after infiltration of Xoo strains. (**b**) Lesions in rice leaves at 14 d after inoculation of Xoo strains. Lesion lengths were presented as means ± standard errors (*n* = 3). Columns with different letters are significantly different according to LSD test (*p* < 0.05).

Role of the glycosyltransferase gene in bacterial virulence was determined by examining the HR in nonhost tobacco and the pathogenicity in host rice seedlings. HR lesions were distinct in tobacco leaves after 24 h of inoculation with the wild-type C2 and the complement c2289 while was indistinct after inoculation with C2R and k2289. No HR appeared in mock inoculation with water (Figure 8a). In rice leaves, the length of lesion was about 13.0 cm after inoculation with C2 and c2289 while only about 5.0 cm after inoculation with C2R and k2289. No lesion appeared in the mock inoculation with water. The virulence of the spontaneous mutant C2R and insertional mutant k2289 was significantly reduced (Figure 8b).

## 4. Discussion

Bacteria can interfere with phage infection at all stages including phage adsorption, DNA entry, DNA replication, transcription and translation, phage assembly and release [5]. Bacteria acquiring resistance to phages through spontaneous mutations is the main driving force for the coevolution of bacteria and phages [6,17]. This study found that the spontaneous frameshift and premature mutation of a gene CDS2289 encoding a glycosyltransferase conferred Xoo resistance to lytic phage X2 by altering LPS production and profiles, bacterial surface morphology and bacterial motility.

The mutation in the gene CDS2289 encoding the glycosyltransferase was the only mutation found in the encoding region in the screening of spontaneous mutants of Xoo resistant to the lytic phage X2. Interestingly, another screening of the Tn5 mutant library of Xoo strain C2 resistant to the phage X2 also found a Tn5 insertion in the same gene CDS2289 (unpublished data) and attracted our intention to study the role of glycosyltransferase in the Xoo–phage interactions. Here, comparing phenotypes of the wild-type strain C2, the spontaneous mutant C2R, the insertional mutant k2289 and the complement mutant c2289 and the almost silenced expression of the mutated gene revealed the function of the glycosyltransferase in LPS production and profiles, bacterial surface morphology and bacterial motility and its roles in phage-resistance and bacterial virulence.

**Figure 9:**
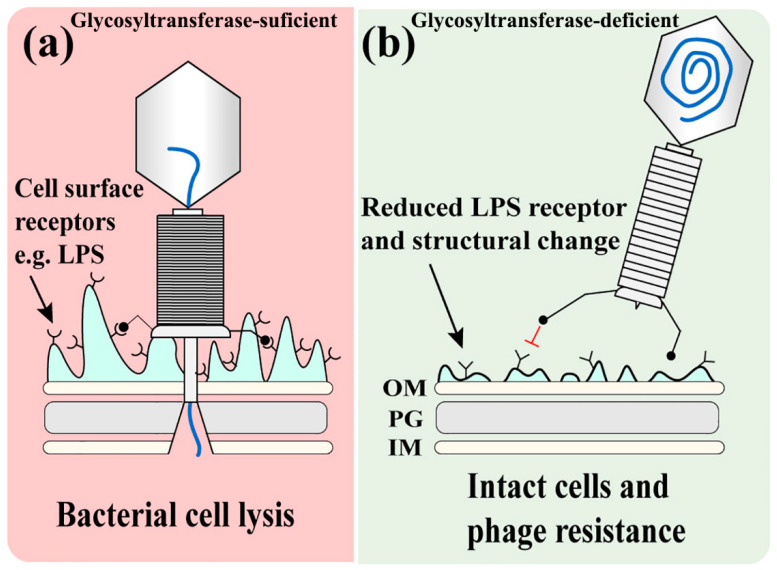
Mechanistic model for glycosyltransferase-mediated initial interaction between bacteria and phages. (**a**) After the phage binds to the host cell surface receptors including LPS, phage contracts and injects phage DNA into bacterial cytoplasm, leading to bacterial cell lysis. (**b**) The mutation of the glycosyltransferase gene reduces LPS receptors and alters bacterial surface structures, leading to the reduced adsorption of phages and the resistance to phages. Red T sign means the Barrier of phage adsorption to bacterial surface.

Glycosyltransferase, which is widely found in bacteria, plays important roles in the etiology of disease and therapeutic targets through catalyzing the formation of glycosidic bond using sugar donors containing a nucleoside phosphate or a lipid phosphate leaving group [38,39]. Glycosyltransferase is involved in the synthesis of LPS, one of the phage adsorption receptors [18] and pathogen-associated molecular patterns [40,41], which is composed of lipid A, the oligosaccharide core and O-antigen [42]. The adsorption of phage ΦL7 onto *Xanthomonas campestris* requires binding to a complex receptor consisting of LPS [43]. In this study, the glycosyltransferase mutants of Xoo reduced phage adsorption efficiency by about 50% and displayed dramatic changes LPS profiles especially in O-antigen region and cellular surface morphology compared with the wild-type Xoo. These results indicate that LPS, especially the O-antigen, which is highly variable compared with the oligosaccharide core having relatively conservative structure [37], is the receptor of the Xoo lytic phage X2. Therefore, we propose a model about the glycosyltransferase-mediated initial interaction between bacteria and phages (Figure 9). Phages bind to LPS, the cell surface receptor, and then inject DNA into host bacterial cells. The mutation of the glycosyltransferase gene in association with the LPS synthesis reduces the amount of cell surface receptors and changes the LPS structure, conferring the bacterial resistance to phages (Figure 9).

In addition to being involved in phage–bacterium interaction, LPS is involved in the interactions between bacteria and plant host as an important surface-associated virulence factor for bacterial pathogens including *Xanthomonas* [44,45,46]. The lack of LPS O-chain led to different host defense responses and reduced virulence in *X. campestris* pv. *campestris* [41,47] and *X. oryzae* pv. *oryzicola* [48]. Similarly, in our study, glycosyltransferase-deficient Xoo mutants showed weak pathogenicity both in non-host plant tobacco and host plant rice compared with the wild-type Xoo because of the change in LPS profile and weakened motility, which is also an important virulence factor in many pathogenic bacteria [49].

Bacterial surface receptors such as LPS face the pressure of lytic phages and host defense and constantly undergo changes and adaptions. Here, the change of Xoo LPS is associated with both the resistance to phage and reduced virulence to plant host. This study on interactions among plant pathogenic bacteria, phages and plants highlights that the reduced virulence to a host is a fitness cost associated with phage resistance, which is reported in many cases of human pathogens [50,51]. The enhanced phage resistance and weakened virulence of Xoo would become a good case for balanced coevolution strategy among phage, bacteria and host plants.

## Figures and Tables

**Table 1:** Strains and plasmids used in this study.

Strains and Plasmids	Description	Source or Reference
*Xanthomonas oryzae* pv. *oryzae*		
C2	pathogen of BLB	Lab collection
C2R	Spontaneous phage-resistant strain of C2	This study
k2289	Km, glycosyltransferase--deficient strain, insertional mutant	This study
c2289	Km, Cm, complementation of CDS2289 gene	This study
*Escherichia coli*		
DH5α	F-Φ80d *lac*ZΔM15Δ(*lac*ZYA-*arg*F) U169 *recA1 endA1*, *hsdR17*(rk-, mk+) *phoAsupE44 λ- thi-1 gyrA96 relA1*	Vazyme, Nanjing, China
S17-1 λ pir	λ Lysogenic S17-1 derivative producing π protein for replication of plasmids carrying oriR6K; *recA* pro *hsdR* RP4-2-Tc: Mu-Km::Tn7 λ− pir	[21]
Plasmids		
pJP5603	Km; R6K-based suicide vector; requires the pir-encoded π protein for replication	[22]
pRADK	Km, Cm; host broad expression vector	[23]

Km and Cm indicate Kanamycin-resistant and Chloromycetin-resistant, respectively.

**Table 2:** Primers used in this study.

Primers Name	Sequences (5′-3′)	Length
Gene knockout primers		
k2289-F	CGGGATCCGCCGGCCTGCACTATCAC	1549 bp
k2289-R	CGGAATTCGGTGCATGCCTTCCACCG	
Gene complementation primers		
c2289-F	CAAGCTCGCGAGGCCTCGAGTCTCGTTGAACTTGCGCGTC	3113 bp
c2289-R	GTGAATCGATAGATCTCGAGTCACTCCGCAATAAACAACGCC	
RT-qPCR primers		
q2289-F	ATGTCGATGTCTTTGGCCGA	195 bp
q2289-R	CGCAAATGCGGCGAACG	
qgyrB-F	CGCCTACCAGGAAACCATGT	120 bp
qgyrB-R	TTCTGCTCGATGTAGGTGCC	

Note: Nucleotides with underline indicated restriction sites of the enzymes: *Bam*HI, *Eco*RI, or *Xho*I.

**Table 3:** Summary of the spontaneous mutation sites in phage-resistant mutant C2R of Xoo strain.

Genetic Variants	Gene	Annotation	Position	Reference Bases	Alternate Bases	Quality
Indel	CDS 949 upstream	Mobile element protein	910,589	AGG	A, AG	572.78
Indel	CDS 1674 upstream	Maltodextrin ABC transporter, ATP-binding protein MsmX	1,639,078	CGG	C, CG	772.39
Indel	CDS 2146 upstream	Cold shock protein of CSP family	2,103,224	CA	C	40,774.04
Indel	CDS 2289	Glycosyltransferase	2,266,307	G	GC	10,079.96
Indel	CDS 4567 downstream	avirulence protein	4,437,505	GC	G	13,253.19
SV	CDS 1	Cysteinyl-tRNA synthetase	10	N	<DUP>	3639.68

## Data Availability

All data supporting the conclusions of this article are included in this article. The CDS2289 gene sequences of Xoo strain C2 have been deposited at GenBank database with Accession No. ON245468.
